# Editorial: Challenges and Conundrums in Cannabinoid-Based Treatments for Epilepsy Syndromes and Associated Neurobehavioral Comorbidities

**DOI:** 10.3389/fnbeh.2021.781852

**Published:** 2021-11-11

**Authors:** Ricardo Gómez-Nieto, Dolores E. López, Norberto Garcia-Cairasco

**Affiliations:** ^1^Institute of Neuroscience of Castilla y León, University of Salamanca, Salamanca, Spain; ^2^Institute for Biomedical Research of Salamanca (IBSAL), Salamanca, Spain; ^3^Department of Cell Biology and Pathology, Faculty of Medicine, University of Salamanca, Salamanca, Spain; ^4^Neurophysiology and Experimental Neuroethology Laboratory, Physiology Department, Ribeirão Preto School of Medicine, University of São Paulo, Ribeirão Preto, Brazil; ^5^Department of Neuroscience and Behavioral Sciences, Ribeirão Preto School of Medicine, University of São Paulo, Ribeirão Preto, Brazil

**Keywords:** antiseizure medications, cannabidiol, cannabinoids, comorbidities, epilepsy, experimental-animal models, clinical epilepsy, seizure

Epilepsy is the one of most common neurological disorder that affects people of all ages. This devastating disease presents a very different etiology and a wide variety of symptoms. Although the hallmark of those symptoms is the recurrent and unprovoked seizures, epilepsy should be considered a spectrum disorder that can lead to many physiological disturbances accompanied by a great diversity of behavioral manifestations. Thus, epilepsy syndromes include not only seizures, but also several comorbid conditions related to behavioral and psychiatric disorders such as cognitive impairments, depression, and anxiety. Despite the availability of numerous and different antiseizure treatments, many fail to manage the uncontrolled electrical activity in the epileptic brain. This becomes a major medical problem, the so-called pharmacoresistant or refractory epilepsy, as it is often a chronic and lifelong condition for many patients. Uncontrolled seizures are linked in both cause and consequence to significant risk of severe brain injuries with irreversible modifications of cerebral organization, which can result in poorly controlled seizures, despite ongoing antiseizure medications, and can even cause death. Pharmacoresistant epilepsy further leads to debilitating psychopathic consequences and many antiseizure substances are found to have a role in exacerbating physical and/or psychiatric symptoms. Thus, it is not uncommon for epileptic patients with pharmacoresistance to also experience several episodes of significant depression and/or anxiety. Bearing in mind that the major goal of epilepsy therapy is for patients to be free of seizures and adverse effects, a reinforced understanding of pharmacoresistance is considered a hot spot in epilepsy research. The availability of newer anticonvulsant components and the development of a promising pipeline of future antiseizure medications is becoming a reality. In this regard, and despite the cannabis usage in ancient times and further criminalization, it is striking the strong interest developed in recent years for the use of cannabis-derived compounds to treat epilepsy syndromes and associated neurobehavioral comorbidities. Among all the phytocannabinoid present in *Cannabis sp*., it is particularly noteworthy the cannabidiol (CBD) that interacts with therapeutic targets in body and brain and exerts a well-defined anticonvulsant profile, without adverse psychoactive effects and abuse liability. The cannabinoid system is overwhelming as is the complex association of many ligands, various types of receptors, multi-signal pathways as well as different ion channels, and furthermore, not all cannabinoids act through the endocannabinoid system. Although previous studies have found that cannabinoids improve seizure control and have benefits on neurobehavioral function, unresolved questions and controversy persist.

This Research Topic aimed to highlight basic and patient-centered research focused on the cannabinoid-based treatments for epilepsy syndromes and associated neurobehavioral comorbidities (Figure 1). This Topic has gathered twelve articles, including three reviews, one brief research report, and eight original research contributions from high-profile scientists in the field. Readers will find high-level information on the multidisciplinary and cutting-edge techniques, including molecular, histological, electrophysiological, pharmacological, and behavioral approaches as well as *in silico* and *in vitro* methodologies. This article collection is meant to provide a comprehensive insight into the therapeutic use of cannabinoids, including the anticonvulsant effects, the involvement of the endocannabinoid system, pharmacoresistance mechanisms, potential interactions with classic antiseizure medications and possible side effects, making a wide variety of key information available to clarify the relevance of cannabinoid-based treatments in refractory epilepsy as well as comorbid anxiety- and depression-like behavior (Figure 1). The first article of this Topic (Auzmendi et al.) reported inhibitory effects of CBD on the active efflux of P-glycoprotein-dependent Rhodamine-123 by using cultures of rat astrocytes and vascular endothelial cells subjected to hypoxia. The outcome of this *in vitro* approach contributes to elucidate how overexpression of P-glycoprotein at the blood-brain barrier level can limit the access of antiseizure medications to the brain parenchyma. Additionally, the authors performed *in silico* studies to predict a possible direct interaction between P-glycoprotein and CBD as a substrate/competitive inhibitor, which supported the use of CBD as an adjuvant therapy in refractory epilepsy. In this context, three original research articles deal with the CBD's mechanism of action and the role of endocannabinoid system in patients with drug-resistant mesial temporal lobe epilepsy (DR-MTLE) and comorbid mood disorders. First, Rocha et al. evaluated the tissue levels of endocannabinoids and the cell signaling transduction after the activation of cannabinoid-receptor-1 in the hippocampus (epileptogenic area) and the temporal neocortex (seizure propagation area) of patients with DR-MTLE, with and without anxiety and depression. The results indicated that enhanced endocannabinoid neurotransmission is involved in the absence of comorbid mood disorders. Consistently, Martínez-Aguirre et al. focused on evaluating the interaction between CBD and 5-hydroxytryptamine-1A receptors in cell membranes obtained from the hippocampus and temporal neocortex of patients with DR-MTLE. The radioligand displacement and GTPγS-binding assays showed that CBD interacts with human 5-hydroxytryptamine-1A receptors, acting as an inverse agonist that might modify neuronal excitation and epileptic seizures in DR-MTLE patients. Complementing these studies, Nuñez-Lumbreras et al. assessed the protein expression levels and Gαi/o protein-induced activation by cannabinoid-receptors-1 and 2 in the brain microvascular endothelial cells of patients with DR-MTLE. The results obtained from the immunofluorescence, Western blot and GTPγS-binding approaches were compared with those from autopsies of non-epileptic patients and correlated with the clinical data. This study revealed differences in the protein expression of cannabinoid-receptors between the hippocampus and the temporal neocortex, suggesting that cannabinoid-receptors with high efficiency represent an important therapeutic target for maintaining the integrity of the blood-brain barrier in DR-MTLE patients. The research article by Morales-Chacón et al. further deals with the impact of CBD on the brain function of patients with pharmacoresistant epileptic encephalopathy who received pharmaceutical-grade CBD (Epidiolex®) as adjunctive antiseizure therapy. The authors used functional connectivity and network topology derived from electroencephalograms, and interestingly found that CBD treatment was related to inhibition of the transition of the interictal to the ictal state as well as the improvement of electroencephalogram organization and brain function. They advocated for using this network analysis approach on electroencephalographic signals to assess the effects of CBD in clinical practice. Seizure disorders are common during childhood and the immature brain is highly susceptible to developing neuronal hyperexcitability under different pathological conditions. In this Topic, the review by Vega-García et al. compiles crucial information from pre-clinical and clinical studies that examine the effects of cannabinoids on epileptogenesis in early life. The authors spotlight the beneficial therapeutic effects of cannabinoids and pointed out methodological limitations such as the small sample size and the short follow-up period in pediatric studies. The efficacy and safety of CBD treatment in pediatric epilepsy was also discussed. In a brief research report, Santos et al. examined the effects of systemic administration and intracerebral microinfusion of several cannabinoid-receptor agonists in two rodent models of epilepsy, the genetically epilepsy-prone rats (GEPR-3) and the *Area Tempestas* model. By assessing and comparing seizure scores and electroencephalograms in baseline, vehicle-treated and treatment conditions of both experimental models, this study add to our understanding of potential sites of action of cannabinoids in the context of putative antiseizure treatment. Since there are strong indications that the endocannabinoid system modulates epileptic seizures by regulating neuronal excitability, it is vital to investigate the expression of cannabinoid receptors in pre-clinical animal models of epilepsy. In this respect, this topic contains two original research articles that determined the protein expression of cannabinoid-receptors-1 in the brain of two well-established genetically audiogenic rodent strains, in which the generalized tonic–clonic seizures are triggered by intense sound stimulation. Lazarini-Lopes et al. used a detailed anatomical analysis to assess the effects of acute and chronic audiogenic seizures on cannabinoid-receptor-1 expression in the hippocampus and amygdala of the Wistar Audiogenic Rat (WAR). Similarly, Fuerte-Hortigón et al. used immunohistochemistry and gene expression analysis to study the differential distribution of the cannabinoid-receptor-1 in the brain of the genetically audiogenic seizure-prone hamster (GASH/Sal). These complementary studies shed light on the relationship between the cannabinoid-receptor-1 and seizure susceptibility in both genetic model of audiogenic seizures, setting it up as a potent regulator of neuronal excitability. In this context, readers might wonder about the anticonvulsant effects of cannabinoids and their synergistic interactions with conventional antiseizure agents in audiogenic seizure models. The review article by Lazarini-Lopes et al. provides an overview on the pharmacological modulation of the endocannabinoid system in audiogenic seizure susceptibility as well as the effects of *Cannabis*-derived compounds, with special attention to CBD. The authors pointed out that the assessment of cannabinoids in epilepsy related comorbidities is an under-explored research field and should be further investigated. To delve deeper into these issues, the article by Cabral-Pereira et al. examined the behavioral and molecular effects of acute and chronic administrations of CBD and valproate on the GASH/Sal audiogenic seizures, as well as the coadministration of both drugs. It was found that CBD slightly attenuated seizure behaviors without adverse effects, and the combination of both drugs did not alter the therapeutic outcome of the valproate monotherapy, which helps prevent the animals from getting convulsions. The effects on the gene expression of protein channel and receptors targeted by the CBD were further explored in the epileptogenic focus. The review article by Medeiros et al. explores the endocannabinoid system as a possible pharmacological landmark for mimicking a form of “on-demand” desynchronization analogous to those proposed by deep brain stimulation in the treatment of epilepsy. The review also discusses the evidence supporting the role of the endocannabinoid system in modulating the synchronization and/or coupling of distinct local neural circuitry, which presents implications on the physiological setting of proper sensory-motor integration.

**Figure 1:**
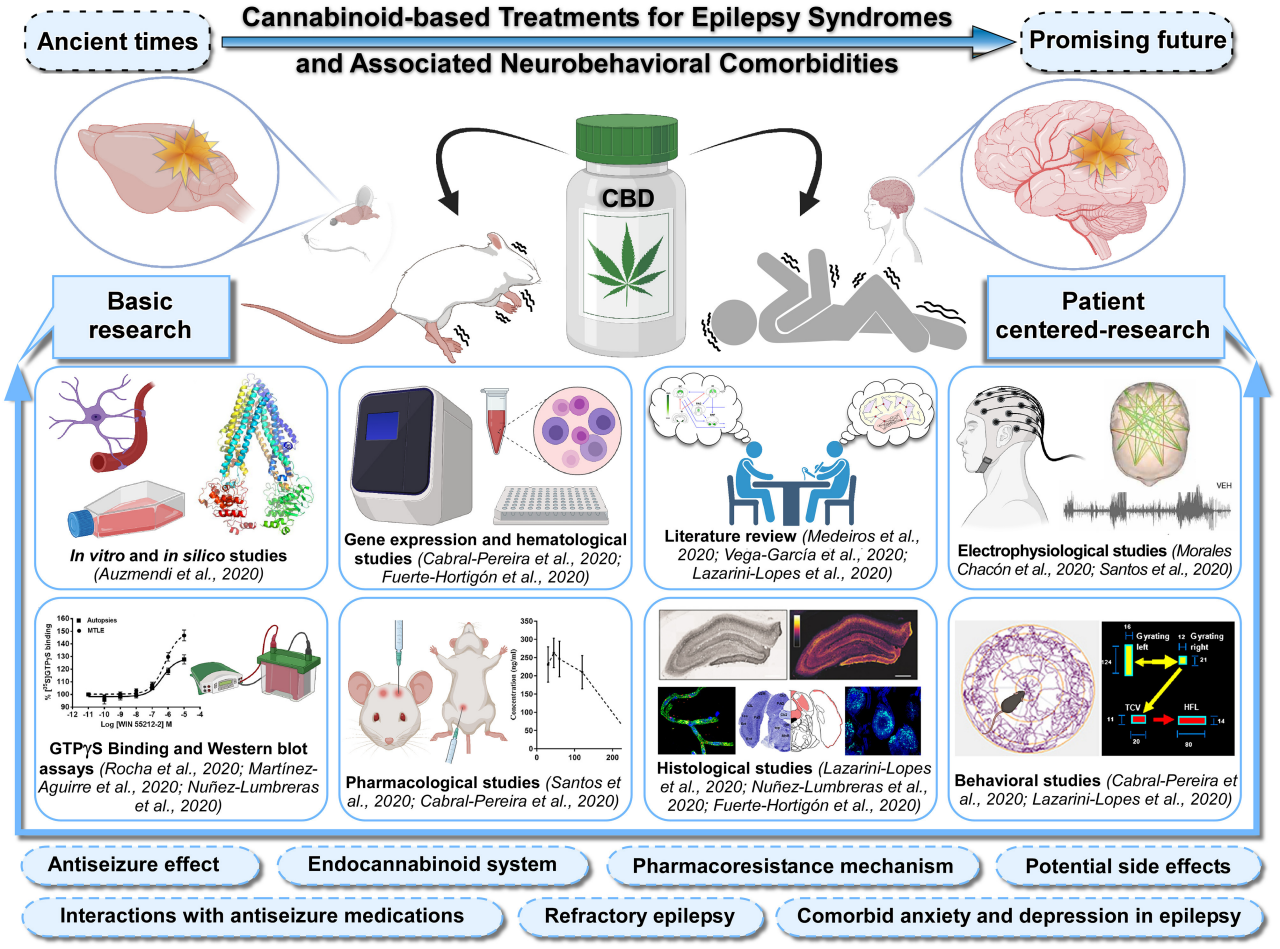
Sketch of the Research Topic's main issues. Advances in the knowledge of cannabinoid-based treatments for epilepsy syndromes and associated neurobehavioral comorbidities should be fostered by greater integration between basic and patient-centered research. This Special Research Topic brings together scientific studies in experimental models and patients with epilepsy using a broad range of cutting-edge techniques, including molecular, histological, electrophysiological, pharmacological, and behavioral approaches as well as *in silico* and *in vitro* methodologies. In addition, it also contains integrative literature reviews that generate new frameworks and perspectives on the topic. This Topic collectively provides a comprehensive insight into the anticonvulsant effects of cannabidiol (CBD), role of the endocannabinoid system, pharmacoresistance mechanisms, potential interactions with classic antiseizure medications, possible side effects, with a view to tackle the use of cannabinoids in refractory epilepsy as well as comorbid anxiety and depression in epilepsy. Articles shown in the figure were grouped by methods and experimental approaches. *Figure created with Canvas Software and icons based on* *Biorender.com*.

To overcome the challenges and conundrums in the cannabinoid-based treatments for epilepsy syndromes at the present time, the investigators worldwide are tirelessly designing and conducting experiments. This Research Topic represents an excellent example of this collaborative effort and commitment. The Topic Editors hope that these thoughtful papers would benefit those researchers to advance in this exciting research field, inspiring novel bench-to-bedside ventures.

## Author Contributions

All authors listed have made a substantial, direct and intellectual contribution to the work, and approved it for publication.

## Funding

This study was supported by the research grant from the Instituto de Salud Carlos III (ISCIII), co-financed with European Union FEDER funds (#PI19/01364, PIs: DL and RG-N), the São Paulo Research Foundation (FAPESP grants: 14/50891-1, 19/05957-8, 19/00849-2, and 2019/02787-4, PI: NG-C), the National Council for Scientific and Technological Development (CNPq grants: 465458/2014-9 and 305883/2014-3, PI: NG-C), the Coordenação de Aperfeiçoamento de Pessoal de Nível Superior (CAPES—Finance code 001, PI: NG-C), and CAPES-Print (Process no. 88887.370299/2019-00, PI: NG-C), as well as the SPRINT-São Paulo Researchers in International Collaboration program from the USAL/FAPESP (#2019/16574-2, PIs: NG-C and DL).

## Conflict of Interest

The authors declare that the research was conducted in the absence of any commercial or financial relationships that could be construed as a potential conflict of interest.

## Publisher's Note

All claims expressed in this article are solely those of the authors and do not necessarily represent those of their affiliated organizations, or those of the publisher, the editors and the reviewers. Any product that may be evaluated in this article, or claim that may be made by its manufacturer, is not guaranteed or endorsed by the publisher.

